# Prolonged febrile seizures cause reversible reductions in white matter integrity^☆^

**DOI:** 10.1016/j.nicl.2013.10.010

**Published:** 2013-10-24

**Authors:** M. Yoong, K. Seunarine, M. Martinos, R.F. Chin, C.A. Clark, R.C. Scott

**Affiliations:** a Neurosciences Unit, UCL Institute of Child Health, 4/5 Long Yard, London WC1N 3LU, UK; b Imaging and Biophysics Unit, UCL Institute of Child Health, 30 Guilford Street, London WC1N 2AP, UK; c Young Epilepsy, Lingfield, Surrey, UK; d Developmental Cognitive Neurosciences Unit, UCL Institute of Child Health, 30 Guilford Street, London WC1N 2AP, UK; e Edinburgh Neurosciences, The University of Edinburgh, Muir Maxwell Epilepsy Centre, Edinburgh, UK; f Geisel School of Medicine at Dartmouth, Department of Neurology, Lebanon, NH, USA

**Keywords:** Diffusion tensor imaging, Epilepsy, Febrile status epilepticus, TBSS

## Abstract

Prolonged febrile seizures (PFS) are the commonest cause of childhood status epilepticus and are believed to carry a risk of neuronal damage, in particular to the mesial temporal lobe. This study was designed to determine: i) the effect of prolonged febrile seizures on white matter and ii) the temporal evolution of any changes seen.

33 children were recruited 1 month following PFS and underwent diffusion tensor imaging (DTI) with repeat imaging at 6 and 12 months after the original episode of PFS. 18 age-matched healthy control subjects underwent similar investigations at a single time point. Tract-based spatial statistics (TBSS) was used to compare fractional anisotropy (FA), mean diffusivity (MD), axial diffusivity (AD) and radial diffusivity (RD) between patients and controls on a voxel-wise basis within the white matter skeleton.

Widespread reductions in FA along multiple white matter tracts were found at 1 and 6 months post-PFS, but these had resolved at 12 months. At one month post-PFS the main changes seen were reductions in AD but at 6 months these had predominantly changed to increases in RD.

These widespread white matter changes have not previously been noted following PFS. There are many possible explanations, but one plausible hypothesis is that this represents a temporary halting of normal white matter development caused by the seizure, that then resumes and normalises in the majority of children.

## Highlights

• Widespread reductions in FA occur in children after prolonged febrile seizures.
• These reductions persist up to 6 months post-PFS but resolve by 1 year.
• This may represent a seizure-related disruption of white matter development.

## 1 Introduction

Convulsive status epilepticus (CSE) is the commonest medical neurological emergency in childhood, and prolonged febrile seizures (PFS) are the commonest subgroup of children with CSE (Chin et al., 2006). Children with PFS have been shown to have hippocampal injury post-PFS (Provenzale et al., 2008; Scott et al., 2003), reduced cognitive performance compared to controls (Hesdorffer et al., 2011; Martinos et al., 2012) and an increased risk of developing epilepsy (Maher and McLachlan, 1995; Verity and Golding, 1991) in later life. However, while a childhood history of PFS is strongly associated with temporal lobe epilepsy with mesial temporal sclerosis (TLE:MTS), prospective evidence to support a causal link to TLE:MTS or other types of epilepsy remains weak (Cendes, 2004).

Some evidence suggests that the damage caused by CSE is not limited to the hippocampus but often affects extra-hippocampal structures (Arfanakis et al., 2002; Bernasconi, 2003). In animal models other cortical and subcortical regions are also affected by CSE (Choy et al., 2010; Sloviter, 2005); furthermore, CSE in humans is associated with widespread changes in cortical grey matter (Milligan et al., 2009) and subcortical white matter tracts (Kim et al., 2001; Okamoto et al., 2006). It seems likely therefore that any deleterious effects of childhood PFS may also affect extra-hippocampal structures, which may be important in the pathogenesis of TLE:MTS or other subsequent epileptic disorders.

Diffusion tensor imaging (DTI) is an MRI technique that can provide information about tissue microstructure in vivo. Indices such as fractional anisotropy (FA), mean diffusivity (MD), axial (AD) and radial diffusivity (RD), have been used as markers of white matter structure and integrity (Beaulieu, 2002; Neil et al., 2002). Tract-based Spatial Statistics (TBSS) (Smith et al., 2006) provides an operator independent, automated technique for performing voxel-wise analysis of diffusion data along a common white matter skeleton.

Most previous studies have either taken place in adults or in the acute phase following PFS. Region of interest (ROI) based DTI studies in adults with established TLE has shown that reductions in FA occur both within the ipsilateral temporal lobe and adjacent white matter tracts, with significant but less pronounced reductions in more remote tracts (Gross et al., 2006; Otte et al., 2012). TBSS based studies of adults with established TLE have also shown decreases in FA in several white matter tracts including anterior corpus callosum, parahippocampal gyrus and temporal gyri (Afzali et al., 2011). However, no studies in children have yet been reported. This longitudinal study was therefore undertaken to investigate the pattern and longevity of white matter abnormalities in children following PFS.

## 2 Materials and methods

### 2.1 Subjects

33 children were recruited from hospitals around North London following an episode of PFS between 1st March 2007 and 1st March 2010. Each child underwent MRI investigations including DTI at 1, 6 and 12 months post-PFS. Detailed clinical information about medical history, developmental history and PFS semiology was collected from each participating child at first appointment. These children formed part of a larger cohort investigating the effects of CSE in childhood whose clinical details have previously been reported (Yoong et al., 2012). A total of 68 children with PFS were notified to the study team during this time period, of which 33 agreed to undergo MRI. Those children whose families did not consent to participate were compared with participants and were found not to be significantly different in age (Mann–Whitney U, p = 0.532) or index of multiple deprivation (IMD, http://www.ons.gov.uk) (Mann–Whitney U, p = 0.813). There were differences in the gender composition of participants vs. non-participants (Chi-squared, p = 0.04), with a greater proportion of females in the participants.

Only limited data regarding seizure semiology was available for non-participants, but from the initial referral data, they did not differ from those who were seen with respect to whether the PFS was focal or generalised (Fisher's exact test, p = 0.47), continuous or intermittent (Fisher's exact test, p = 0.529) or whether the parents reported pre-existing developmental problems in the child (Fisher's exact test, p = 0.25). Reported mean seizure duration was significantly shorter for those who did not take part compared to those who did (50 min vs. 72 min, Mann–Whitney U, p = 0.009). Clinical characteristics are summarised in Table 1.

Enrolled children were seen at Great Ormond Street Hospital for Children, London (GOSH) for an assessment including clinical review and MRI investigations 1 month after the PFS. MRI investigations were performed with the child awake, in natural sleep, under sedation or under general anaesthesia as required, depending on the age and developmental stage of the child. These investigations were repeated at 6 and 12 months after the initial episode of CSE.

An age-matched group of 18 children, comprising a mixture of healthy volunteers and patients recruited from hospital clinics with no history of seizures and structurally normal MRI on routine clinical reporting, was recruited for use as controls. 3 children in this group had isolated hearing loss with no other neurological abnormalities and the remainder were neurologically normal.

All volunteers were scanned either awake or during natural sleep. Patients received sedation or anaesthesia as required by the requesting physician. Children with intracranial lesions described on their clinical report were excluded from the control group. Each control only had a single MRI investigation as: 1) repeating the general anaesthesia for those children who were having MRI for clinical reasons would not have been appropriate and 2) repeating an MRI scan on those young controls that had their MRI during post-prandial natural sleep after one year would have required sedation.

### 2.2 Image acquisition

All images were acquired on a 1.5 T Siemens Avanto MRI system (Erlangen, Germany). An imaging protocol used for the evaluation of children with epilepsy and optimised for the visualisation of mesial temporal structures was carried out, including a T1-weighted 3D-FLASH sequence: TR = 4.94 ms, TE = 11 ms, acquisition matrix 256 × 224, in-plane resolution 1.0 × 1.0 mm, slice thickness 1 mm. Additional diffusion-weighted echo planar imaging was acquired: TR = 96 ms, TE = 2700 ms, acquisition matrix 96 × 96, in-plane resolution 2.5 × 2.5 mm, slice thickness 2.5 mm. Diffusion-weighting was performed along 20 non-collinear directions using maximum b values of 1000 s/mm^2^. One acquisition with no diffusion-weighting (b_0_ image) was included with each set of diffusion-weighted sequences. The scan was repeated three times to improve SNR and data merged together without averaging. Subjects who moved during one or more of the acquisitions had it repeated until 3 good acquisitions were achieved, if this was not possible then they were excluded from analysis. *TractoR* scripts (http://code.google.com/p/tractor/, (Clayden et al., 2011)) were used to generate FA and MD maps according to standard formulae (Basser, 1995; Pierpaoli and Basser, 1996). AD and RD maps were calculated from DTI eigenvalues using *fslmath*. Each scan was reviewed during MRI acquisition and scans with significant motion artefact discarded.

### 2.3 TBSS

TBSS was performed using standard scripts contained within the FSL software package (Smith et al., 2004). All subjects' FA data were aligned into a common space using the nonlinear registration tool FNIRT (Klein et al., 2009). TBSS was then used to perform a search for the most representative image in our cohort and each subject was aligned to that image before being affine-transformed onto the 1 × 1 × 1mm MNI52 template brain. This was done separately for each time-point. Each subject was then visually inspected to check the validity of the registration. Registration was deemed acceptable for all subjects and none were removed from the analysis at this stage.

A mean FA composite image was created by averaging all the warped FA images and thinned to create a mean FA skeleton representing the centres of all tracts common to the group thresholded at FA > 0.2. Each subjects' aligned FA data was then projected onto the mean skeleton and analysed using voxel-wise cross-subject statistics.

The *randomise* tool from FSL was used to perform voxel-wise two-sample nonparametric permutation tests (Nichols and Holmes, 2002) with 5000 permutations on the aligned white matter skeleton to detect differences in FA between children with PFS and controls. Threshold-free cluster enhancement (Smith and Nichols, 2009) was used to detect significant clusters of voxels and correct for multiple comparisons. A parallel TBSS analysis performed comparing patients' scans at 1 and 6 months with a paired *t*-test showed that FA increases with age over the entire white matter skeleton in children with PFS as has previously been shown in healthy controls (Provenzale et al., 2010; Wolff et al., 2012). Therefore gender, age and intracranial volume (ICV) were entered as covariates and coded as nuisance variables. The results were stored as statistical maps with p < 0.05 taken as the level of significance. Results were visualised as colour-coded masks and superimposed on the MNI52 template brain. Localisation of differences was performed with a reference white matter atlas (Melhem et al., 2002).

As longitudinal data was not available for control subjects, each of these stages was repeated for each time point, thus 3 separate cross-sectional TBSS analyses were made comparing patients at each time point with controls. As the age of the patients changed over the study, a subset of control subjects was chosen from our cohort to approximate the age range of the patients at each time point; each comparison was made to a different, albeit overlapping set of control subjects. Each analysis was also repeated against the entire control cohort to demonstrate that no bias had been introduced due to use of a subset of controls for each cross-sectional analysis, with similar results.

MD, AD and RD maps were subjected to the warps generated from the alignment of the FA maps and a similar analysis performed to identify areas of significant difference.

The mean white matter skeleton generated during the TBSS analysis was used as an ROI to measure the mean FA, MD, AD and RD over the entire skeleton in each subject before comparison with univariate ANOVA between control and patient groups at each time point. Age, ICV and gender were entered as co-variates in concordance with the TBSS analysis. Adjusted values were determined after correction for age/ICV/gender.

## 3 Results

### 3.1 Demographics

32/33 children with PFS had imaging suitable for use in this analysis at at least one time point. The remaining child did not tolerate DTI and was excluded. There were 29 children with suitable imaging at 1 month, 17 at 6 months and 19 at 1 year post-PFS. 11 children had imaging at all 3 time points and a further 11 had suitable imaging at two time points. 18 children from the control group tolerated DTI and were in a suitable age range. Ages of all of the children are summarised in Table 2. There were no significant differences in age (Mann–Whitney U, p > 0.1) or gender composition (Chi-squared, p > 0.5) between patient and control groups. No significant differences were found between those who had 1, 2 or 3 scans in: age at CSE, duration of CSE, seizure focality or whether the seizure was continuous or intermittent.

### 3.2 Reduced white matter FA in children following PFS

**Fig. 1:**
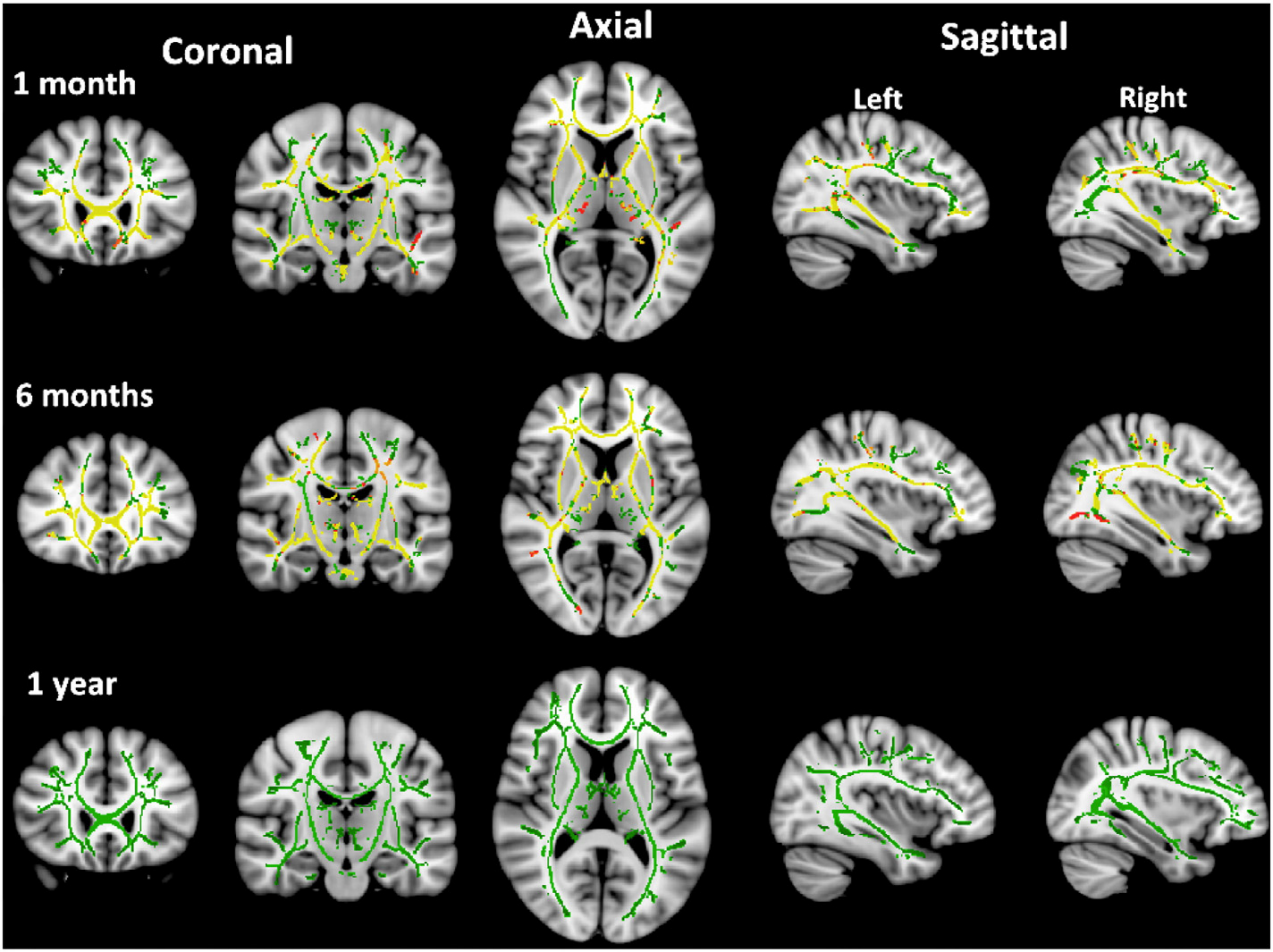
Maps of fractional anisotropy at 1, 6 and 12 months post-PFS. Red-Yellow areas show areas of significant reduction compared to control subjects. The white matter skeleton is outlined in green.

The results of the TBSS analysis comparing FA between patient and control groups are shown in Fig. 1. There were widespread bilateral reductions in FA at 1 month post-CSE in across the majority of major white matter tracts including corpus callosum, internal/external capsule, inferior fronto-occipital fasciculus and anterior corona radiata. These reductions were largely still present at 6 months post-CSE. However by 1 year post-CSE there were no longer any significant differences between patients and controls. A summary of the tracts found to be involved at each time point is given in Table 4. There were no areas found with significantly higher FA in patients than controls at any time point.

**Supplementary Fig. 1:**
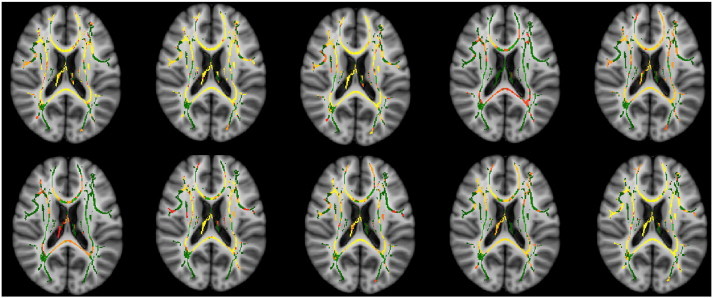
Axial slices from each of 10 repetitions of TBSS analysis at 1 month post-PFS. Each repetition used a subset of 17/32 patients with PFS and the same 15 controls. Red-yellow areas show areas of significant increase over control FA values.

In order to avoid potential bias from the greater number of patients scanned at 1 month post-PFS, the TBSS analysis at this time point was repeated 10 times using 10 different, randomly chosen subsets of 17 patients and the same 15 controls. Similar reductions in FA were obtained in each repetition (Supplementary Fig. 1).

In order to avoid potential bias from the greater number of patients scanned at 1 month post-PFS, the TBSS analysis at this time point was repeated 10 times using 10 different, randomly chosen subsets of 17 patients and the same 15 controls. Similar reductions in FA were obtained in each repetition (Supplementary Fig. 1).

**Fig. 2:**
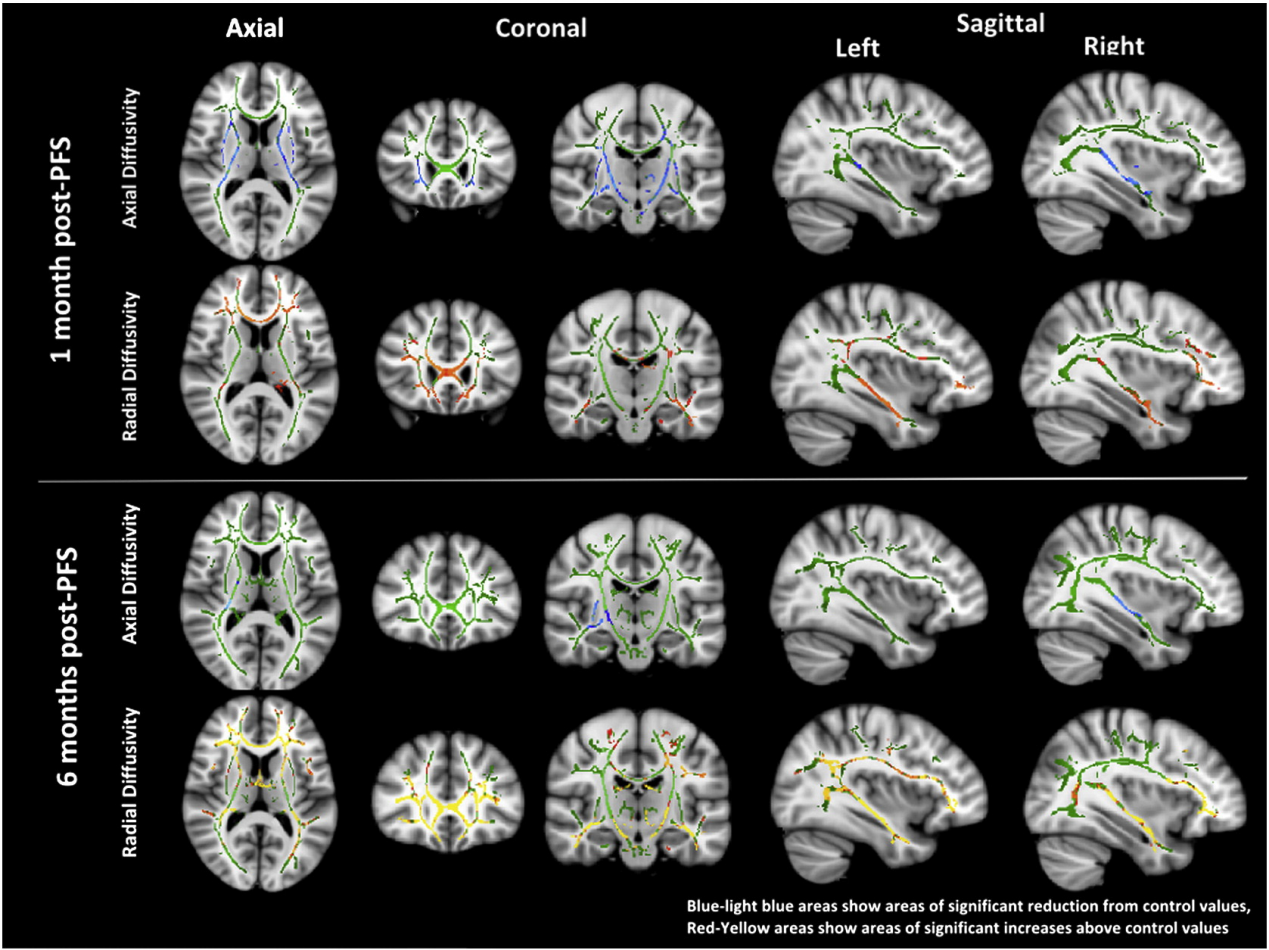
Maps of radial and axial diffusivity at 1 and 6 post-PFS.

There were no significant differences in MD found between patients and controls at any time point. Fig. 2 shows the results of the analysis of AD and RD at 1 and 6 months. At 1 month there was a mixture of reduced AD in the anterior thalamic radiation, fronto-orbital fasciculi and internal/external capsule and increased RD in the corpus callosum and fronto-occipital fasciculi. However at 6 months, the reductions in AD had mostly disappeared, whereas the RD increases covered the majority of the white matter skeleton. At 1 year there were no significant differences in AD or RD between controls and patients.

### 3.3 Whole skeleton DTI

**Fig. 3:**
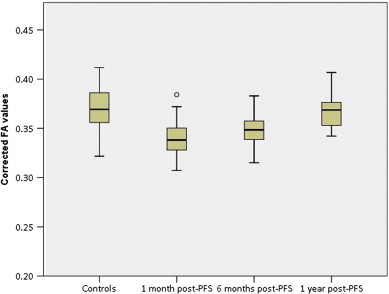
Corrected mean FA values for patients and controls.

Mean values for FA, MD, AD and RD over the entire white matter skeleton for each of the groups are given in Table 3. FA values over the entire TBSS skeleton were significantly below control values at 1 month (p = 0.039) and 6 months (p = 0.027) post-PFS, but had normalised by 1 year. These can be seen in Fig. 3. MD was not significantly different at any time-point. No significant differences were found after correction for multiple comparisons in AD or RD although there was a trend towards lower AD at 1 month and higher RD at 6 months post-PFS, but no differences in either at 1 year.

## 4 Discussion

### 4.1 Diffusion changes following PFS

Widespread reductions in FA were detected in multiple white matter tracts following PFS using TBSS analysis. These changes were apparent at 1 month post-PFS, and remained until at least 6 months post-PFS. However, by 1 year they appeared to have resolved. This reduction in FA appeared to be predominantly caused by decreases in AD at 1 month, and by increases in RD at 6 months post-PFS. No differences were detected in MD on TBSS analysis.

Similar changes were seen in mean FA over the entire white matter skeleton, with significant reductions in FA seen at 1 and 6 months, before normalisation at 1 year. No significant differences were found in mean AD or RD averaged across the skeleton, although there was a trend towards decreased AD at 1 month, and increased RD at 6 months post-PFS, possibly reflecting the regional nature of changes in these metrics.

### 4.2 Interpretation

Excluding isolated case reports (Gong et al., 2008; Kassem-Moussa et al., 2000; Okamoto et al., 2006), there have been no systematic studies looking at diffusion metrics following CSE to date. Reported cases have shown acute changes in cortical grey and sub-cortical white matter on DWI, with a reduced apparent diffusion coefficient (ADC) (Kassem-Moussa et al., 2000; Okamoto et al., 2006) and persistent reductions in fornix FA (Gong et al., 2008). The acute MRI changes have generally been observed to resolve by 1 month post-CSE, although the majority of cases reported had residual neurological problems and associated chronic MRI changes. These reports are not representative of the general population of children with CSE, as persistent neurological abnormalities only occur in a minority (Yoong et al., 2012) and represent the most severe outcomes.

Two MRI studies of children post-PFS reported increases in hippocampal volume (Scott et al., 2003) and T2 hyper-intensities (Provenzale et al., 2008), suggestive of transient and self-resolving oedema (Scott et al., 2003; Scott et al., 2006).

A recent meta-analysis of DTI studies of TLE showed that while the largest differences are seen in ipsilateral temporal lobe and adjacent white matter, significant reductions are also found in contralateral temporal lobe and remote tracts (Otte et al., 2012). There have been consistent reports of reduced FA in corpus callosum, external capsule, ipsilateral temporal lobe and frontal white matter tracts. These studies of adults with established TLE, many years after any initial precipitating episode, represent the final end point of changes that would be expected if PFS was causally linked with TLE:MTS.

None of our patients has been diagnosed with epilepsy, nor do they show visible changes on MRI or gross neurological abnormalities (Yoong et al., 2012). While reductions in FA were found in the areas highlighted in previous adult studies of TLE, children with PFS also showed more widespread FA reductions than those reported in TLE.

The first scans on this cohort were 1 month post-CSE, when acute oedema was expected to have resolved. This time point was deliberately chosen to avoid potential confounding from resolution of the acute changes that are known to occur following PFS (Scott et al., 2006). One possibility is that the DTI changes represent longer-term seizure-related neuronal damage such as myelin breakdown or axonal degeneration (Beaulieu, 2002; Song et al., 2003). However, given the relatively benign functional outcomes found in these children (Shinnar et al., 2008; Martinos et al., 2012) severe brain injury seems unlikely and 6 months is a prolonged period for recovery from a milder injury. More plausibly, a disruption of the normal maturational processes of the developing brain would also produce reduced FA and increased RD in comparison with a normally maturing control population (Geng et al., 2012) with subsequent catch-up growth leading to normalisation at 1 year.

Another possibility is that these abnormalities pre-exist the PFS and relate to the predisposition to prolonged seizures in response to febrile illnesses. If this increased susceptibility is related to aberrant white matter development, this could underlie the widespread diffusion changes. Febrile seizures have a large genetic component to their aetiology (Nakayama, 2009) and are most common between 6 months–5 years, with a peak incidence at 18 months (Nordli, 2010). As susceptibility decreases with age, it might be expected that any causative structural abnormality also resolves, although the mean patient age at the end of this study was 3 years, which is still within the window of susceptibility.

### 4.3 Limitations

There are a number of limitations that need to be addressed when considering the results of this study. Most importantly, as ethical approval was not given to collect longitudinal data from control subjects, a true longitudinal comparison was not possible, necessitating sequential cross-sectional comparisons. This approach has less power to detect true differences, especially in the same patients over time. Despite this, significant changes were observed in children with PFS while restricting our analysis to a whole group level. Decreases were also seen in the age-adjusted mean FA in the patient group, implying that this was not an artefact of the TBSS analysis.

Another limitation is the proportion of eligible patients who did not agree to participate. While we have shown that, from the information available, they were not significantly different from participants other than in reported seizure duration, as this data was not collected consistently for non-participants, it is not possible to completely exclude the possibility of an undetected bias. It is possible that children who had a more severe PFS were more likely to agree to take part, although the data from non-participants is of necessity less reliable. Regardless even if this were the case it would not materially alter our conclusions.

Thirdly there was a significant drop out rate; with only 11/32 patients receiving scans at all three time points. The majority of patients did however receive scans at 2 time points and there were no significant differences in age, gender or seizure semiology between those who did and did not continue to participate at each stage.

Finally TBSS only detects changes across the white matter skeleton and would not detect isolated hippocampal damage such as MTS. Furthermore, the hypothesised timeframe for the development of MTS is several years after PFS, outside the scope of this study. Directly addressing the link between PFS and MTS will require longer term, hippocampal-specific data.

Even given these limitations however, this study has revealed novel information regarding the impact of PFS on structural brain development, with potential longer term implications.

## 5 Conclusions

This is the first systematic study of diffusion changes following PFS in children, demonstrating several novel findings. There appear to be widespread reductions in FA following PFS which are still present at 6 months post-PFS, but normalise by 1 year post-PFS. We have advanced several hypotheses for these observations: namely that they represent seizure-induced injury, with subsequent recovery; that they represent a pre-existing structural abnormality related to febrile seizure susceptibility; or alternatively that they represent a seizure-related temporary disruption of white matter development. We suggest that the latter hypothesis offers the most biologically plausible explanation given the nature and timescale of the changes seen but further investigation will be required to provide definitive proof.

The following are the supplementary data related to this article.

Supplementary data to this article can be found online at http://dx.doi.org/10.1016/j.nicl.2013.10.010.

## Funding

This study was funded by the Wellcome Trust (Grant number: 060214⁄HC⁄RL⁄MW⁄kj). This work was undertaken at GOSH⁄University College London Institute of Child Health, which received a proportion of funding from the Department of Health's National Institute for Health Research Biomedical Research Centres funding scheme.

## Disclosure of financial interests

Dr Yoong, Dr Seunarine, Dr Martinos and Dr Clark report no disclosures. Dr Chin held a National Institute for Health Research Academic Clinical Lectureship and received travel grants from GlaxoSmithKline, Janssen-Cilag, Esai, UCB Pharma and has received an honoraria from Viropharma. Dr Scott is supported by GOSH Children's Charity and has received travel grants from Glaxo-SmithKline, Janssen-Cilag, UCB Pharma, and SPL Ltd.

## Figures and Tables

**Table 1:** Clinical characteristics of all referred children.

	Participants	Non-participants
Number of children	33	35
Male:female ratio	10:23	20:15
Median age at PFS (IQR, range)	1.68 (1.31–2.78, 0.76–4.56)	1.27 (1.03–2.50, 0.84–6.48)
Mean seizure duration in minutes (range)	71.7 (30–190)	50.2 (30–130)^a^
Focal onset	5 (15%)	3 (8%)
Continuous seizure activity	21 (64%)	19 (54%)
Previous febrile convulsions	12 (36%)	8 (23%)
Previous PFS	2 (6%)	2 (6%)
Previous developmental problems	5 (15%)	2 (6%)

a Information only available from 27/35 non-participants.

**Table 2:** Age ranges and numbers of children at each time point.

	Number	Male:female ratio	Median age (years)	Age range (years)
Patients 1 month post-PFS	29	9:20	1.86	0.85–4.61
Patients 6 months post-PFS	17	6:11	2.25	1.19–5.01
Patients 1 year post-PFS	19	5:14	2.90	1.76–5.45
Controls	18	8:10	2.45	0.62–5.47
Controls used for 1 month group	15	7:6	2.32	0.62–4.28
Controls used for 6 months group	12	5:7	2.62	1.69–5.25
Controls used for 1 year group	14	5:9	2.90	1.69–5.47

**Table 3:** Mean adjusted diffusion metrics across the white matter skeleton and comparison with control values.

Skeletal FA, MD, AD and RD adjusted for age, ICV and gender (95%CI)								
	Mean FA	p-Value	Mean MD	p-Value	Mean axial diffusivity	p-Value	Mean radial diffusivity	p-Value
Controls	0.364 (0.354–0.375)		0.899 (0.884–0.915)		1.276 (1.261–1.290)		0.711 (0.692–0.730)	
Patients 1 month post-PFS	0.346 (0.337–0.355)	0.039^a^	0.904 (0.890–0.918)	NS	1.256 (1.243–1.269)	0.135	0.732 (0.716–0.748)	NS
Patients 6 months post-PFS	0.343 (0.337–0.355)	0.027^a^	0.915 (0.897–0.932)	NS	1.266 (1.249–1.282)	NS	0.740 (0.719–0.760)	0.147
Patients 1 year post-PFS	0.356 (0.345–0.368)	NS	0.896 (0.878–0.913)	NS	1.260 (1.244–1.276)	NS	0.714 (0.693–0.734)	NS

p-Values represent significance levels for differences from control values and are adjusted for multiple comparisons.

a p < 0.05 after Bonferroni correction for multiple comparisons.

**Table 4:** Major white matter tracts showing significant changes in diffusion metrics at 1 and 6 months post-PFS.

Time following PFS	Decreases in FA		Decreases in AD		Increases in RD	
	1 month	6 months	1 month	6 months	1 month	6 months
Anterior thalamic radiation (left + right)	X	X	X			X
Inferior fronto-orbital fasciculus (left + right)	X	X	X			X
Anterior corona radiata (left + right)	X	X	X			X
Genu of the corpus callosum	X	X			X	X
Body of the corpus callosum	X	X			X	X
Splenium of the corpus callosum	X	X			X	X
Internal capsule	X	X	X			X
External capsule	X	X	X	Right only		X
Inferior fronto-occipital fasciculus (left + right)	X	X	X	Right only	X	X
Uncinate fasciculus (left + right)	X	X	X		X	X
